# Young-Onset, ROS1-Rearranged Adenocarcinoma of the Lung With Cardiac Tamponade: A Case Report

**DOI:** 10.7759/cureus.71777

**Published:** 2024-10-18

**Authors:** Masafumi Nojiri, Ryudai Abe, Sumito Nagae, Takuya Tanaka, Yutaka Takahara

**Affiliations:** 1 Respiratory Medicine, Kanazawa Medical University, Ishikawa, JPN

**Keywords:** cardiac tamponade, entrectinib, pulmonary embolism (pe), ros1, young-onset lung adenocarcinoma

## Abstract

Adenocarcinoma with ROS1 rearrangement is rare. Carcinomatous pericarditis is generally seen in advanced stages and is a life-threatening condition. A 21-year-old male was admitted to our hospital because of severe dyspnea due to cardiac tamponade identified on echocardiography. A diagnosis of ROS1-rearranged adenocarcinoma of the lung was made from the pathological and gene mutation analyses of the cell block from his pericardial fluid. Oral administration of entrectinib was highly effective, and his serum carcinoembryonic antigen (CEA) level improved from 247 to 10.8 ng/mL. The present case has clinical significance because a pathological and genetic diagnosis was made from the pericardial effusion fluid, and the clinical manifestations of cardiac tamponade due to lung cancer were well controlled by the administration of entrectinib.

## Introduction

Rearrangement of ROS1 is a rare gene mutation in lung cancer, found in about 1% of non-small cell cancers [1]. Carcinomatous pericarditis is seen in advanced stages in about 2% of cancer patients [2]. Hemodynamic disruption and cardiac tamponade occur, especially when large amounts of fluid are retained. The course is often fatal, and cardiac tamponade associated with lung cancer is considered to have a very poor prognosis. Since carcinomatous pericarditis is often seen in the advanced stage, it is very rare to encounter it as the first manifestation [3]. To the best of our knowledge, this is the first report of ROS1-rearranged lung cancer with cardiac tamponade as the initial manifestation.

## Case presentation

**Figure 1:**
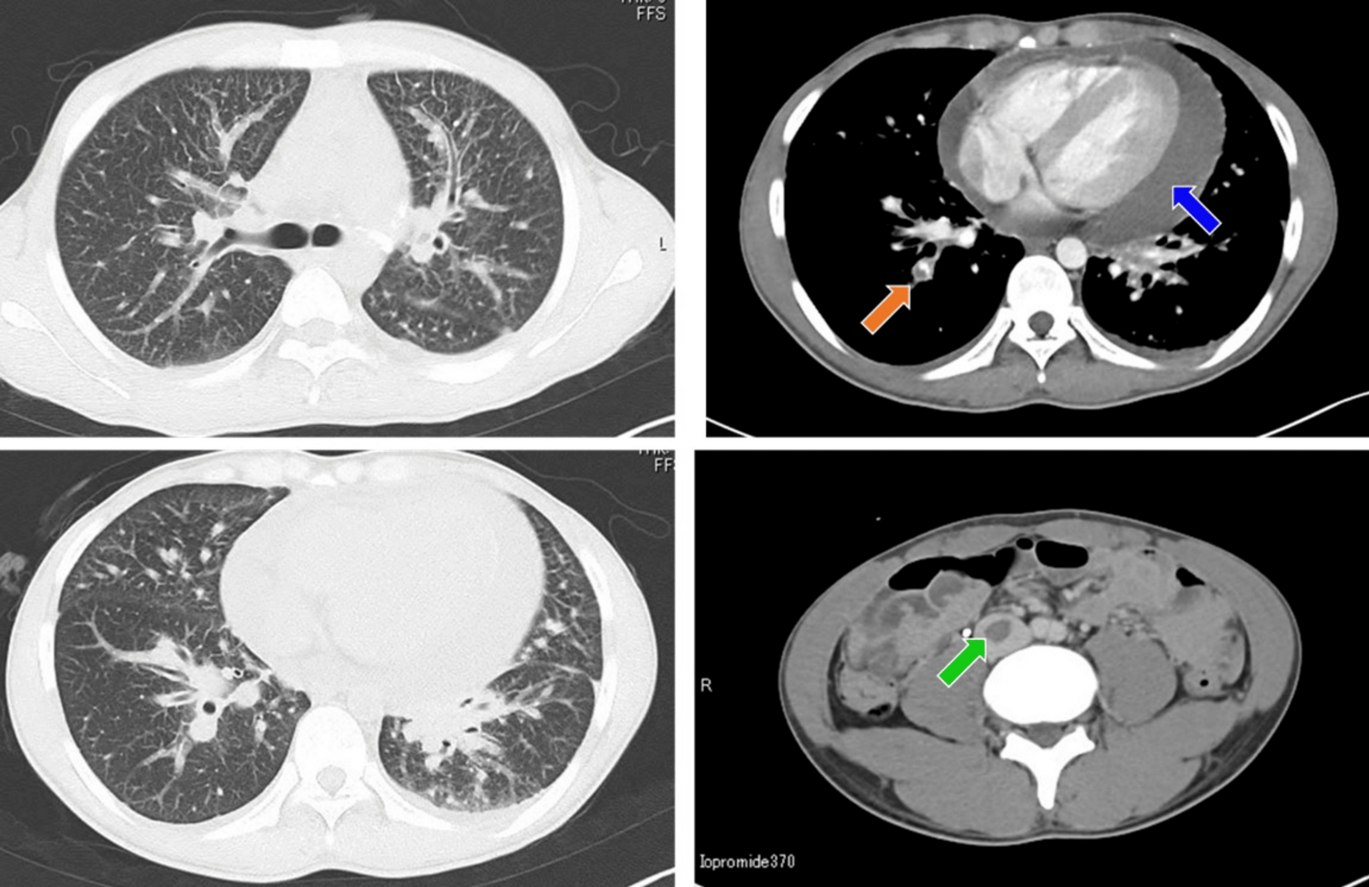
Chest CT Chest CT showing cardiac tamponade (blue arrow), pulmonary emboli (orange arrow), and deep vein thrombosis (green arrow). CT: computed tomography

A 21-year-old Japanese male visited a nearby clinic with a one-month history of worsening dyspnea (modified Medical Research Council (mMRC) grade 2). His chest radiograph showed marked cardiomegaly, and he was then referred to our hospital on the same day. There were no other symptoms such as cough, sputum, hemoptysis, or pleuritic chest pain. He had no chest pain and no past medical or drug history. He was a never-smoker, and there was no family history of lung cancer. On physical examination, his height was 163.5 cm, weight was 53.5 kg, temperature was 36.8°C, blood pressure was 108/60 mmHg, radial pulse was 106/minute and regular, and SpO_2_ was 96% on room air. Coarse crackles were heard on auscultation of bilateral lung bases. On cardiac auscultation on admission, S1 and S2 were normal, with no murmurs. There was no leg edema. On laboratory examination, hemoglobin (Hb) was 12.5 g/dL (normal range: 14-18 g/dL), C-reactive protein (CRP) was 0.94 mg/dL (normal range: <0.5 mg/dL), and D-dimer was 24.3 μg/mL (normal range: <1 µg/mL). His chest radiograph showed marked cardiomegaly and consolidation in bilateral lower lungs. Chest computed tomography (CT) showed a large pericardial effusion and bilateral lung infiltration, especially in the lower lobes. Contrast-enhanced CT showed thrombi at the right pulmonary artery and inferior vena cava, probably derived from deep vein thrombosis (DVT) (Figure 1). However, no obvious tumor was identified in his lungs.

**Figure 2:**
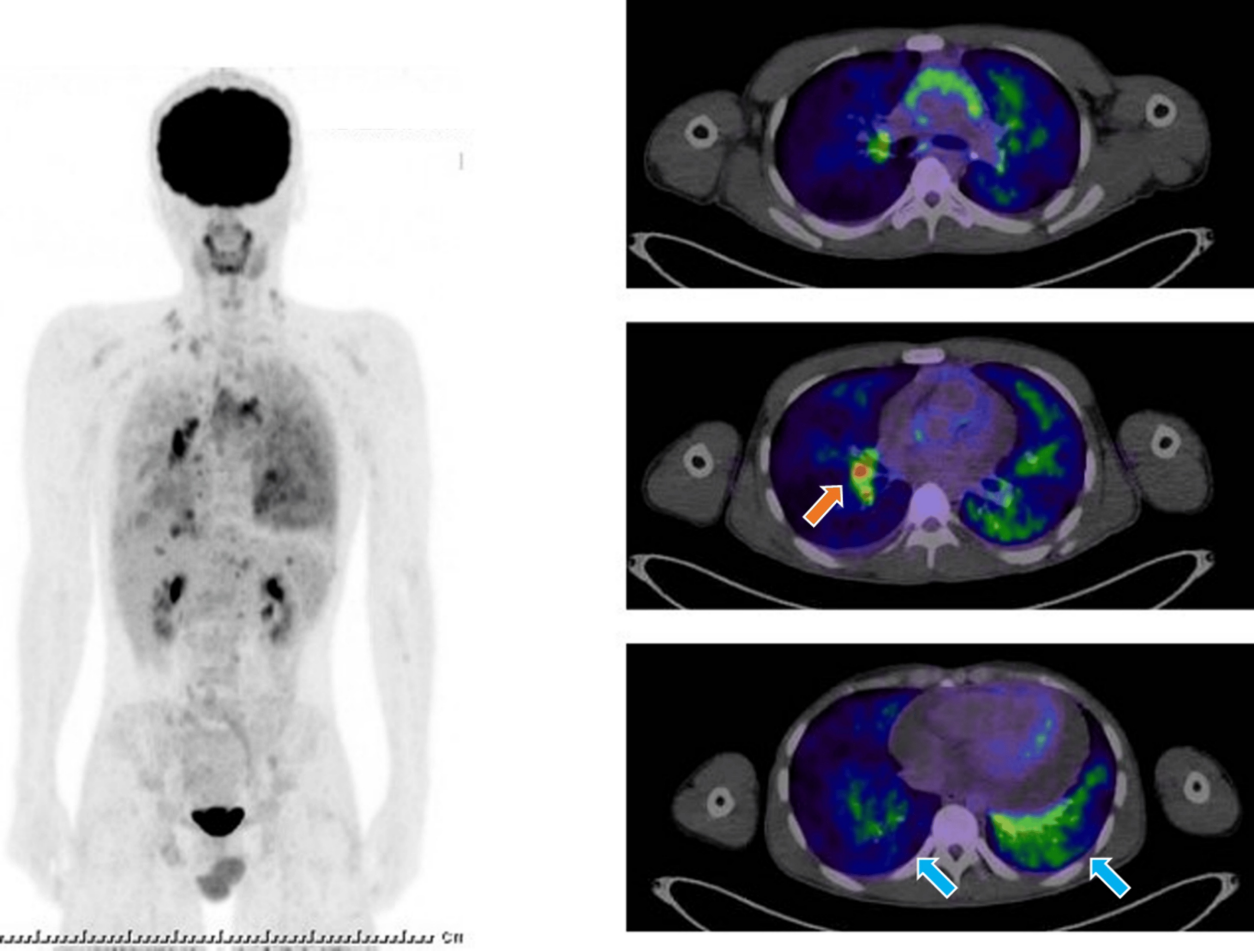
Positron emission tomography Positron emission tomography highlighting both lower lobes (blue arrow) and the lymph nodes (orange arrow).

**Figure 3:**
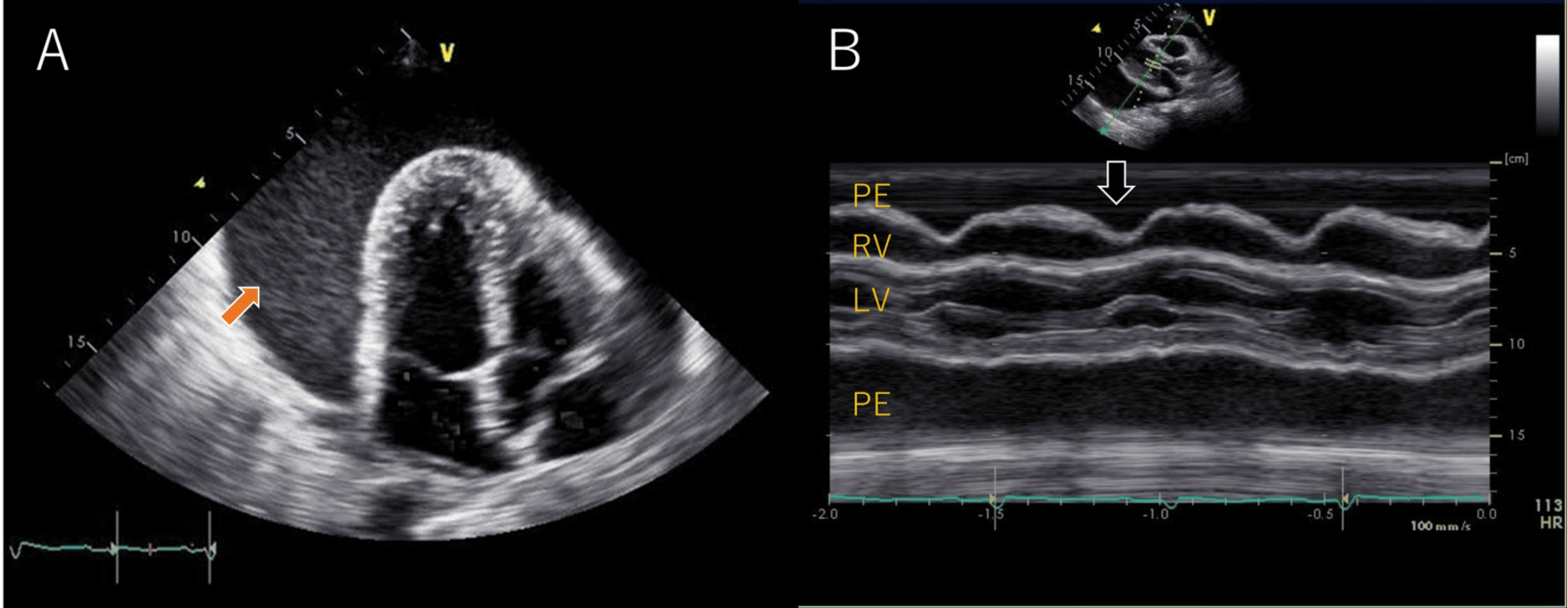
Echocardiography on admission A: Apical four-chamber view echocardiogram demonstrating features characteristic of cardiac tamponade (orange arrow). B: M-mode of diastolic right ventricular collapse in the parasternal long-axis view. Black arrows indicate the early diastolic collapse of the right atrium. PE: pericardial effusion, LV: left ventricle, RV: right ventricle

**Figure 4:**
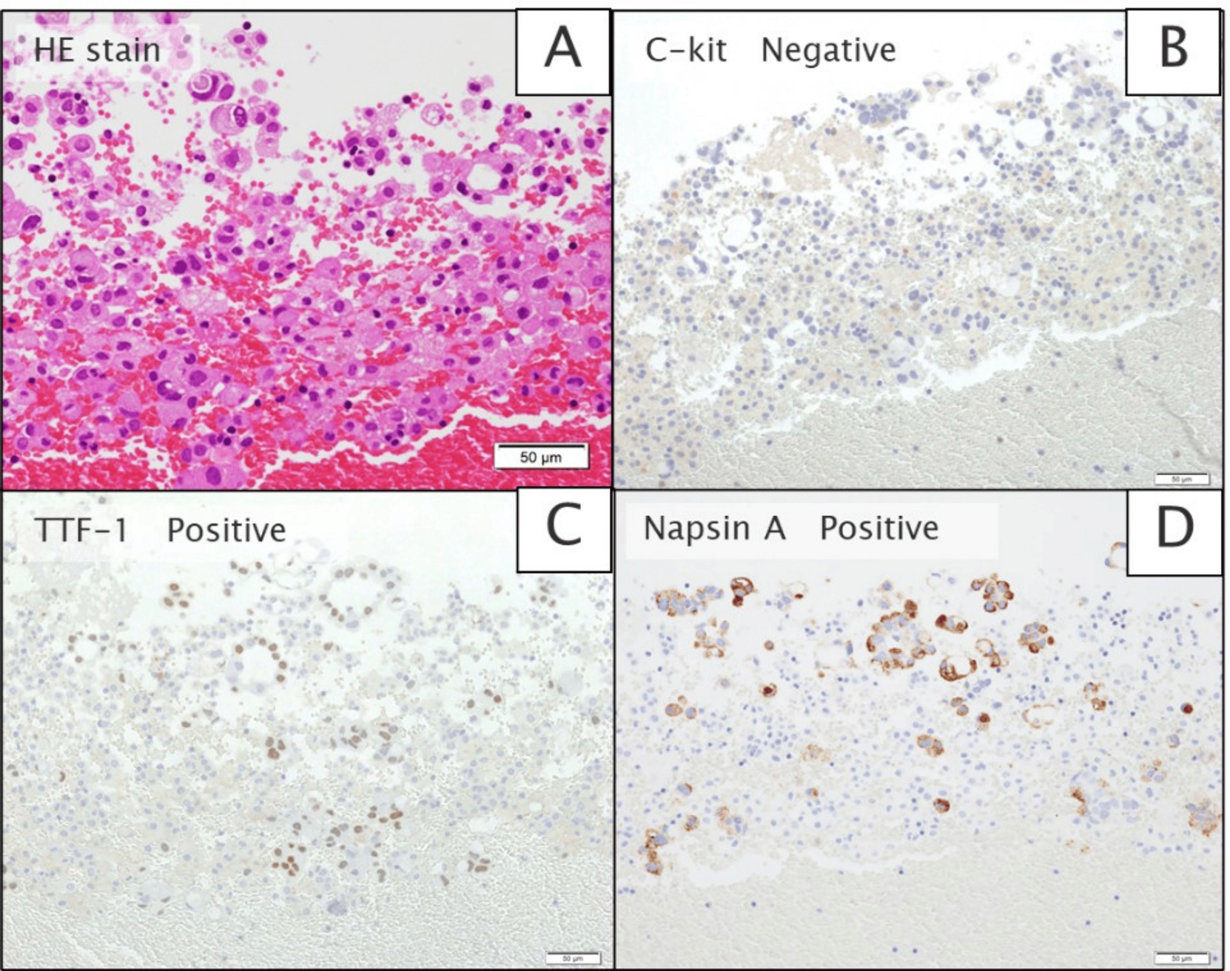
Pathological examination of cell block Cell block showing (A) hematoxylin and eosin staining, (B) negative C-kit, (C) positive TTF-1, and (D) positive Napsin A. TTF-1: thyroid transcription factor-1

Fluorodeoxyglucose (^18^F)-positron emission tomography (FDG-PET) showed diffuse FDG accumulation in the infiltrating shadows of both lower lobes (Figure 2). There was also FDG accumulation (standardized uptake value (SUV): 5.46) in the lymph nodes, indicating possible lymph node metastasis, but no other distant metastases. Cranial magnetic resonance imaging (MRI) showed no brain metastasis. Ultrasound examination showed a massive pericardial effusion, right atrial retraction, and cardiac tamponade (Figure 3). Echocardiography-guided pericardiocentesis was performed, and 1,000 mL of hemorrhagic pericardial fluid was drained. The serum carcinoembryonic antigen (CEA) level, evaluated as a marker for possible cancerous pericarditis, was 404 ng/mL (normal range: <5 ng/mL); such elevation suggested the possibility of cancerous pericarditis. Therefore, the collected pericardial fluid was submitted for pathological examination. After the pericardiocentesis, his dyspnea and respiratory symptoms improved immediately. Adenocarcinoma cells were detected in the pericardial fluid, and ROS1 gene rearrangement was found on examination of the cell block prepared from the same pericardial effusion using next-generation sequencing (Oncomine Comprehensive Assay v3; Thermo Fisher Scientific, Waltham, MA) (Figure 4).

Initially, the possibility of infectious diseases such as viral pericarditis, autoimmune diseases, or endocrine diseases was considered, but since cancer cells were detected in the pericardial fluid, the diagnosis of cancerous pericarditis was made.

He underwent heparinization and placement of an inferior vena cava (IVC) filter. Anticoagulant therapy with edoxaban 30 mg/day was initiated, bearing in mind his bleeding risk in the chest.

**Figure 5:**
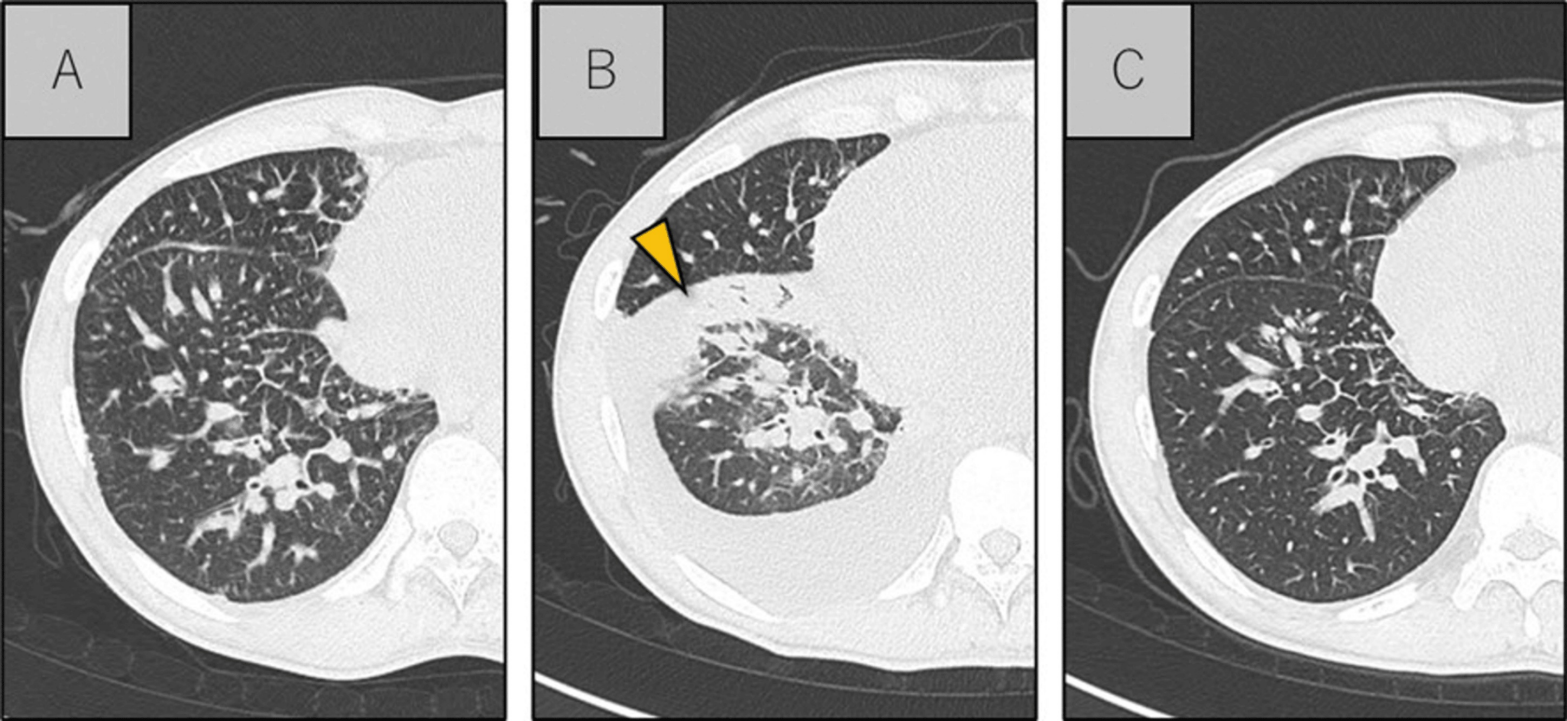
Progress shown on CT imaging A: Before starting crizotinib. B: On day 10 after the start of administration, pneumonia appears (yellow arrow). C: Pneumonia improves five days after the discontinuation of crizotinib. CT: computed tomography

**Figure 6:**
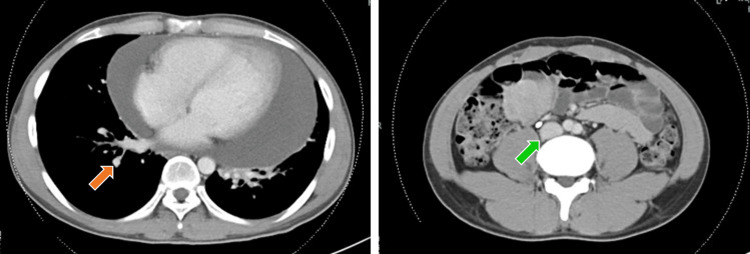
Contrast-enhanced CT of the chest Chest CT showing that the pulmonary emboli (orange arrow) and DVT (green arrow) disappeared on day 84 after the start of entrectinib. CT: computed tomography

After anticoagulation therapy, crizotinib was given orally (twice a day, 250 mg tablet each time). After 10 days of crizotinib treatment, fever, eosinophilia (eosinophils: 7.4%, 325/µL), increased CRP (2.62 mg/dL), and hypoxemia developed. The chest CT showed consolidation of the right lower lobe. Based on these findings, drug-induced pneumonia due to crizotinib was diagnosed (Figure 5). Crizotinib was discontinued, and steroid therapy was not given. Two days after crizotinib was discontinued, his fever and oxygen status improved. A week after crizotinib was discontinued, he was treated with oral entrectinib (600 mg once daily). Six days after starting entrectinib, he developed grade 3 neutropenia and grade 1 hyperuricemia (Common Terminology Criteria for Adverse Events (CTCAE) version 5.0). These treatment-related adverse events (TRAEs) improved one week after withdrawal of entrectinib. Entrectinib treatment was restarted with a reduced dose of 400 mg once daily. During this treatment, hyperuricemia was observed, but it improved with the administration of allopurinol 100 mg. Serum CEA levels improved from 247 to 10.8 ng/mL. The bilateral consolidations were due to cancerous lymphangiopathy, which disappeared after the start of entrectinib and his dyspnea disappeared. However, the pericardial effusion showed only minor improvement. After anticoagulation therapy, the pulmonary emboli and DVT disappeared (Figure 6), and the IVC filter was removed on day 92. The patient has continued entrectinib for more than seven months with no progression of the lung cancer.

## Discussion

Several oncogenic driver alterations are known to occur in lung cancer. ROS1 plays a major role in the activation of several signaling pathways associated with differentiation, proliferation, cell growth, and survival [4]. The resulting ROS1 fusion kinases are constitutively activated and drive cellular transformation [5].

A ROS1-rearranged mutation is a rare gene mutation found in about 2% of lung adenocarcinomas [1]. Juvenile cancer is generally defined as cancer occurring between 15 and 39 years of age at the time of initial cancer diagnosis [6]. Its incidence is 0.37% in the Japanese population, making it extremely rare [7]. In young people, the development of cancer is less affected by smoking and environmental factors [8]. Since juvenile lung cancer is reported to have a high probability of gene mutation, the presence or absence of gene mutation is very important in young patients with lung cancer [9]. The present case is an extremely rare case of ROS1-rearranged lung cancer with cardiac tamponade as an initial manifestation. It has been reported that malignant pericardial effusions are found in 2.7% of all cancer cases, one-third of which are derived from lung cancer [2]. Woo et al. reported that compared to epidermal growth factor receptor (EGFR)-mutant and anaplastic lymphoma kinase (ALK)-rearranged adenocarcinomas, ROS1-rearranged adenocarcinomas were less likely to have distant metastases and more likely to have pericardial metastases and lymphangitis [10]. However, it has also been reported that cardiac tamponade is extremely rare as an initial symptom of lung cancer [3].

Cardiac tamponade is a pathological condition in which increased intrapericardial pressure causes right atrial collapse and reduced cardiac output. It was previously reported that cardiac tamponade has a poor prognosis, with a median survival of approximately three months or less [11-13]. However, longer-term survival is possible in some patients after successful systemic therapy, such as molecular targeted therapy [2]. In the present case, a bronchoscopic approach was difficult because there was no obvious mass in the lung field. Diagnosis and analysis of the ROS1-rearranged mutation were performed using the pericardial fluid, and molecular targeted therapy was started promptly. Mutational analysis of malignant pericardial fluid has been reported to have a higher concordance rate with tissue-based analysis [14]. Pericardiocentesis is essential for performing cytopathological and in cases where a tissue sample might prove difficult to obtain.

In the present case, the patient had DVT and pulmonary thromboembolism. ROS1-rearranged lung cancer is considered to have a high risk of thrombosis. It has been reported that compared with patients with wild-type non-small cell lung cancer, patients with ROS1-rearranged lung cancer are about three times more likely to develop thromboembolism, and the incidence of thromboembolism peaks six months after onset; the cumulative incidence of thromboembolism up to the third year is about 50%, and the incidence of thrombosis after its first episode is low [15]. This is in line with the present case, in which DVT and pulmonary thromboembolism were observed at the first visit, and no new thrombus was observed after treatment.

## Conclusions

A case of ROS1-rearranged lung cancer with malignant cardiac tamponade was described. Cardiac tamponade generally has a poor prognosis. However, cytopathological and mutational analyses of the pericardial fluid confirmed the diagnosis of ROS1-rearranged lung adenocarcinoma, and an adequate therapeutic effect was obtained by oral administration of entrectinib. Pericardiocentesis is essential for cytopathological diagnosis when tissue samples are difficult to obtain, and patients with ROS1-rearranged lung cancer can expect dramatic improvement with treatment, making it worthwhile to aggressively attempt tissue diagnosis in young cancer patients.
